# Real-Time Quality Control of Heat Sealed Bottles Using Thermal Images and Artificial Neural Network

**DOI:** 10.3390/jimaging7020024

**Published:** 2021-02-03

**Authors:** Samuel Cruz, António Paulino, Joao Duraes, Mateus Mendes

**Affiliations:** 1 Polytechnic of Coimbra, Coimbra Engineering Academy, R. Pedro Nunes, 3030-199 Coimbra, Portugal; a21250113@isec.pt (S.C.); jduraes@isec.pt (J.D.); 2 Polytechnic of Coimbra, Higher School of Technology and Management, R. General Santos Costa, 3400-124 Oliveira do Hospital, Portugal; antonio.paulino@estgoh.ipc.pt; 3 Centre for Informatics and Systems, Uiversity of Coimbra, Polo II, Pinhal de Marrocos, 3030-290 Coimbra, Portugal; 4 Institute of Systems and Robotics, University of Coimbra, Rua Silvio Lima, Polo II, 3030-290 Coimbra, Portugal

**Keywords:** quality-control, machine learning, computer vision, thermal images, artificial neural networks

## Abstract

Quality control of heat sealed bottles is very important to minimize waste and in some cases protect people’s health. The present paper describes a case study where an automated non invasive and non destructive quality control system was designed to assess the quality of the seals of bottles containing pesticide. In this case study, the integrity of the seals is evaluated using an artificial neural network based on images of the seals processed with computer vision techniques. Because the seals are not directly visible from the bottle exterior, the images are infrared pictures obtained using a thermal camera. The method is non invasive, automated, and can be applied to common conveyor belts currently used in industrial plants. The results show that the inspection process is effective in identifying defective seals with a precision of 98.6% and a recall of 100% and because it is automated it can be scaled up to large bottle processing plants.

## 1. Introduction

The control of the quality of bottle sealing is a very important step during bottled product’s processing line to assure that the products are safe to transport and store. This applies to numerous products, including food, beverages, detergents, disinfectants and pesticides among other examples. The assurance of the seal effectiveness is particularly relevant when products are potentially harmful to humans or the environment. Thus, processes to automate the bottle seal quality are very important and many techniques have been proposed, using various approaches.

The present paper describes an approach to assess the quality of bottle seals using infrared technology and machine learning. An InfraRed (IR) camera is used to take a thermal picture of the cap after the bottle passes the thermal camera where the bottle cap is sealed. Image processing techniques are used to extract the circle corresponding to the hottest area of the cap, and an Artificial Neural Network (ANN) is used to classify the circle and thus determine the quality of the seal. Bottles having defective seals are identified as they are coming out of the production line and rejected later in the conveyor belt where they are transported. This approach can be implemented using commonly available technology and components and has a low operational costs.

### 1.1. Case Study

**Figure 1:**
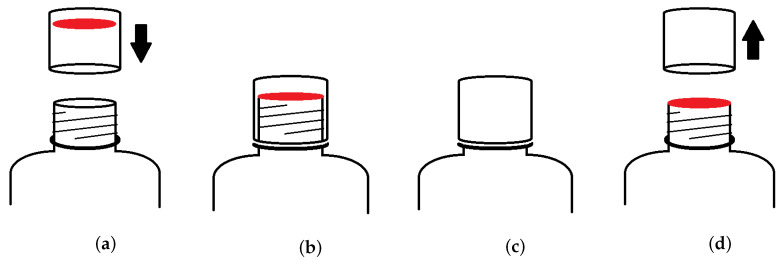
Details of the sealing process. (**a**) The plastic cap contains an aluminium sheet with glue. (**b**) After the bottle is capped, the aluminium circle is glued to the bottle opening. (**c**) Both the seal and the cap should remain in place until the bottle is opened for use. (**d**) Once the plastic cap is removed, the aluminium seal should remain in place until broken.

We applied our approach to a bottled pesticide production plant. The bottles are filled and sealed in a conveyor belt following a process common to most mass production lines:

1. The bottle goes through a liquid filling machine where it is filled with a given substance (in our case it is a toxic pesticide;
2. The bottle goes through a capping machine, where a robotic arm places and attaches the cap to the bottle. Inside the cap there is a thin aluminium circle covered with hot glue, as shown in red in Figure 1a;
3. The capped bottle goes through a thermal camera where the glue is hotted and melted binding the aluminium sheet to the bottle, effectively sealing it, as shown in Figure 1b.

Figure 1a shows the position of the aluminium seal inside the lid as it is being placed on top of the bottle opening to close it. Once closed (as shown in Figure 1b), the seal is fixed and the glue should be evenly spread across the sealing surface. Once the bottle is closed, the seal is unreachable to direct inspection because the lid is made from opaque plastic (Figure 1c). The aluminium seal must remain in place even if the plastic cap is removed, as shown in Figure 1d.

The plastic cap can appear in its correct place even if the seal underneath in broken or incorrectly placed. Because once the bottle is capped the seal is invisible to a human observer, it is difficult to visually inspect the bottle and assess the quality of the seal. However, since the sealing process uses hot glue, it is possible to obtain an infrared image that can be used to infer the state of the seal.

### 1.2. Method Proposed

Our method to assess the quality of the sealing is based on classifying the geometry of the circle imprinted in the thermal image taken right after the bottle leaves the thermal camera that melts the hot glue.

After being cleaned of the background noise, we create sums of the horizontal and vertical image lines to assemble an image signature. Then the signatures are fed to a classifier trained using a machine learning algorithm. The output of the classifier is either good or bad, determining the acceptance/rejection of that particular bottle.

The results of the classification are logged and the logs can later be analysed by the user to inspect the results, or, more important, to improve the classifier, should that need arise. The user is given the option to improve the classifier by selecting wrongly classified images and reclassify them, so the algorithm can be adjusted in order to increase its performance over time. The entire process is controlled by a system that includes a database of the images for analysis and possible retraining, a user interface, the interface to the image processing libraries, the logic for later interfacing with a rejection actuator for online control during the operation of the conveyor belt, and the interface to the machine learning module implementing the classifier. The most relevant part of the system is the image processing and the machine learning model, which is implemented using a neural network.

### 1.3. Paper Structure

Section 2 presents an overview of the state of the art and related work. In Section 4 the architecture of the system is detailed. Section 3 presents a description of the methodology. Section 5 explains how the data are collected, the essential steps of the process, and how it is used in the present study. Section 6 describes the machine learning algorithm. Section 7 presents the results. Section 8 further analyzes the results when compared to the state of art and presents a discussion on the merits and flaws of the methodology and proposed system. Section 9 summarizes and concludes the paper.

## 2. Literature Review

In this section we address the concepts and the works most relating to our own. These include infrared spectroscopy, condition monitoring, classification, non-destructive testing, sealing checking, machine learning, monitoring systems ad architectures, and current quality control systems.

### 2.1. Spectroscopy

Infrared spectroscopy and Near-Infrared Spectroscopy are techniques that analyze the infrared radiation emitted by warm objects that enable the identification of the material properties based on the energy emitted. The basis of these techniques is the fact that materials radiate energy differently accordingly to their specific physical properties.

Asaduzzaman et al. [1] used infrared technology to assess the quality of milk samples. The goal of that work was to identify milk that was falsely labeled as having been produced in ecologically clean mountain areas. That work used raw milk samples from three different farms located in the same geographical region but with different properties such as altitude and farming methods, meaning that the characteristics of the samples differ one from another. After the samples were warmed, their infrared emission wavelengths were analyzed and it was possible to identify the component characteristics and their concentration in the milk. A machine learning algorithm based on k-nearest neighbor was then used to classify the samples based on the characteristics identified.

M. Boiret & F. Chauchard [2] used near-infrared spectroscopy multipoint measurements to control both the distribution and the content of active pharmaceutical ingredients within final drug products [2], in order to create a real-time quality control system. That technology was chosen because it can perform at high speed, it is free from pollution, and it doesn’t require reagents nor sample preparation, plus it is non-destructive and capable of providing information about components existing in Active Pharmaceutical Ingredients (API) tablets [2]. The study used two different batches of tablets manufactured with the same pharmaceutical processes but with different batches of API [2]. Those measurements were done at high-speed, using a conveyor belt system which allowed the analysis of multiple tablets and the data obtained were then analyzed according to their quality (API distribution per tablet) and quantity (quantity of the API content within the final drug products).

### 2.2. Condition Monitoring

Pontes et al. [3] used infrared technology to detect hot locations on a motor in order to identify situations of probable overheating. The goal was to raise the lifespan of the equipment by monitoring changes in the materials’ properties, aiming to detect and fix problems as soon as possible. The images were first preprocessed, filtered and converted to grayscale. Image signatures were created using image line and column sums and the resulting two vectors were then fed to the neural network. Through the comparison of signatures it was possible to evaluate the state of the motor—if the image presented higher values on the signature it meant the motor was overheating. The authors also mention that the whole process required low computing power, thus being possible to implement in a small industrial computer or a raspberry pi.

Elgargni et al. [4] proposed a system that assesses the condition of cutting tools before their use. The authors retrieved multiple visible wavelength and infrared images of the cutting tools and then, after using image processing functions and algorithms to isolate and extract the features, the images were classified using principal component analysis and an artificial neural network. They concluded that the infrared data, combined with suitable image processing and artificial intelligence, could be a very efficient method for on-line condition monitoring of cutting tools.

Haider et al. [5] present another use of infrared to monitor the condition of electric components. In their work the infrared technology is used to obtain images of electrical systems to identify problems in order to reduce maintenance costs, improve productivity and increase machine availability. The method is based on the fact that most components tend to show an increase in temperature while malfunctioning. In that method, instead of using gray scale images, the infrared images are converted to HSV (Hue, Saturation, Value) and processed with different threshold methods such as *Roberts Edge Thresholding*, *Prewitt Edge Thresholding* and *Otsu Thresholding*. Linear Discriminant Analysis (LDA) is then used for the classification and feature dimension reduction. In their experiments they used infrared images of switches, circuits, fuses, and other electrical equipment of their own institution building. A total of 40 sample images were captured, from which 60% were used for training and the remainder for testing. Approximately 55% of the images corresponded to equipment in abnormal condition and 45% in healthy condition. From the tests, the authors concluded that 0.70 was the suitable threshold and then were able to get a model with 100% accuracy.

### 2.3. Object Classification

Zheng et al. [6] propose the implementation of infrared technology to detect and identify battle targets such as cars, trucks and tanks. Despite methods already existing to process images, some army infrared sensors can only obtain a low-resolution image that, once used as input, causes small targets to disappear from the image. The solution to this problem was to design a new model based on the You Only Look Once (YOLO) Convolution Neural Network (CNN), which is a real-time object detection deep learning model (YOLO Official Website: https://pjreddie.com/darknet/yolo/). YOLO, in its third version YOLOv3 uses a 53 layer neural network trained on Imagenet (Darknet-53). It is designed for visible light image detection and recognition and requires a higher resolution, so the authors designed a new model based on this structure. Three models were built: the first one was trained using only visible light images, the second one using infrared images and the last one was first trained with visible images and then fine-tuned with infrared images. The third model not only presented the best results from the group but also presented better results when comparing with the YOLOv3.

### 2.4. Non-Destructive Testing

Dua & Mulaveesala [7] use Infrared Thermal Wave Imaging (ITWI) to evaluate the condition of reinforced concrete in structures. Reinforced concrete is commonly used in the building industry due to its low cost, high strength, robustness and sustainability, along with the ready availability of raw materials. But it also has some drawbacks such as poor tensile strength and ductility [7]. Those drawbacks can lead to the formation of cracks where over time, the ingress of chloride ions and carbon dioxide in the steel surface originates corrosion and leads to the decrease of the cross-section in the material. When that happens, the safety and stability of the structure can be compromised. ITWI is used to measure the heat emitted by the surface of an object. In that study, the object is the surface of the reinforced concrete It is analyzed using ITWI and the images obtained are then processed in order to generate an image where the faults in the material are clearly visible.

### 2.5. Sealing Checking

Al-Habaibeh et al. [8] developed a new mechatronic approach to assure food is correctly sealed inside containers using a laser sealing process, and used infrared thermography combined with image processing to verify the quality of the seal. The infrared camera obtains images of the seal during and after the sealing process. The heat pattern is clearly visible in the resulting images. The thermographical properties are analyzed and if any abnormal area is detected, then the seal is compared to previous faults/problems cases, so that the laser properties can be adjusted. The system is capable not only of detecting the localized heat pattern but also the contamination between the film and the container, as well as the cooling behavior of the materials.

Al-Habaibeh & Parkin [9] use a low-cost infrared sensor to obtain images of several manufacturing processes such as drilling, grinding, welding and soldering. The images contain the distribution of heat in the working machine used in the process and are then evaluated using a novel detection algorithm which is a self-learning approach that distinguishes between normal and faulty conditions of the manufacturing processes. A faulty process will overheat the machine and it can be detected using infrared technology. The systems aims to distinguish between a machine working in normal conditions and overheating machines. This approach was then tested in the four processes mentioned above and the authors concluded that it is most successful with the grinding process and the welding process was the least successful.

D’huys et al. [10] present a pulsed-type active thermography experiment to detect solid contaminants in between seals of heat-sealed flexible food packages. In the study, the authors prepared 30 seal samples contaminated with ground coffee particles that were recorded shortly after sealing. The system obtains high resolution digital images using active IR thermography. These images are then processed using six methods:

1. A method based on a single frame;
2. A method based on a first order polynomial fit of the cooling profiles in the logarithmic domain;
3. A method based on thermal signal reconstruction;
4. A method based on pulsed phase thermography;
5. A method based on principal component thermography;
6. A method based on a 3D matched filter.

The method based on a fit of the cooling profiles presented the lower detection limit meaning that particles with an equivalent diameter of 0.60 mm were detected with a probability of 95%. Also, the detection capabilities of this method do not depend strongly on the time after sealing at which recording of the thermal images was started, making it a robust and generally applicable method.

Shuangyang [11] present a high-speed and high accuracy quality inspection of food packing seals. At the end of a correct sealing process, continuous sealing lines can be seen on the package. First, an image of the seal is taken and then the second step is to threshold the seal zone, making it a black and white image where the continuous seal lines should be highlighted. The authors then use a template to match the image and by comparing the number of pixels matched the system classifies the seal as faulty or acceptable. The system presents an average inspection accuracy of 93.6%.

### 2.6. Machine Learning

Multi-layer perceptron (MLP) neural networks have been used before for image classification. Del Frate et al. [12] assess and optimize neural networks’ performance for pixel-based classification of high resolution satellite images. The images were obtained from two sources: Quickbird data, characterized by very high spatial resolution, and the Landsat data characterized by high spatial resolution. The study aims to distinguish among areas made of artificial coverage (sealed surfaces), including asphalt or buildings, and open spaces such as bare soil or vegetation. The feature extraction and information discovery on urban areas can be used to monitor changes and urban growth over time, which is helpful to improve the environment and safety measures. The neural network obtained presented a satisfactory performance, with the overall accuracy of 87% for Quickbird and 82% for Landsat subareas.

Del Frate et al. [13] describe the application of m-arcsinh, a modified version of the inverse hyperbolic sine function in machine learning algorithms distributed in the Python library scikit-learn. The algorithms used were Support Vector Machines (SVM) and MLP Artificial Neural Networks. The study used six datasets from the scikit-learn and nine datasets from The University of California at Irvine (UCI) ML repository. The aim of the study was to develop a novel computationally efficient and reliable kernel and activation function and evaluate it against the standard functions available in the Python library scikit-learn. The accuracy score and f1 measure were the metrics used to assess the classifiers’ performance. The results showed that using the m-arcsinh kernel and activation function the MLP presented the best classification performance on 10 out of 15 datasets evaluated. The MLP performed better than the SVM, except for two out of 15 datasets. The models’ reliability was higher and better than some standard functions.

Subhadip Basu et al. [14] present a Multi Layer Perceptron based classifier for recognition of handwritten Bangla alphabet. The Bangla alphabet is the second most popular written and spoken language in the Indian subcontinent. The MLP based classifier needs to distinguish between 76 elements. The variations in writing styles of individuals make recognition of handwritten characters difficult and demand the development of a classifier that has generalization abilities. 10,000 alphabetic characters were collected from 200 subjects with different sex and age from which 80% were used as training set and 20% as testing set. The images were scaled to 64 × 64 pixels and converted to binary through thresholding. Only one hidden layer, with 60 neurons, was used, in order to keep a low computational power requirement. The classifier showed performances of 86.46% in the training set and 75.05% in the test set.

### 2.7. Monitoring System Architectures

Dimitris Mourtzis et al. [15] propose an approach for condition-based preventive maintenance of machine tools and cutting tools, based on real-time shop floor monitoring. The data used in the study come from two data sources:

- The multi-sensory system—hardware used to monitor the tools.
- The machine tool operator input—information collected from the operators through reports made on mobile devices.

The data are stored in a database in a cloud service and then the variables under analysis are calculated. The result can be accessed by the maintenance department through a web application. The communication between the operator and the maintenance department is facilitated through the cloud. The use of mobile technology leads to a reduction of the time it usually takes to solve maintenance problems on the factory floor. The authors tested the approach on five milling machines that over a month raised 20 reported problems, which were solved in 4 man hours instead of the predicted 10 man hours, according to the maintenance department.

P. Vithu & J.A. Moses [16] review methods of evaluating the quality of pre-processed food grains using a machine vision system that, using image processing and image analysis techniques, will perform a physical inspection that gives the most information about the condition of the grain. It reviews non-destructive, non-contact and non-invasive methods to evaluate the existence of foreign matters, insect infestations, microbial infections, or grain grain discolouration. Those systems identify and classify the grain according to type and variety. The paper also clarifies the limitations of the use of machine vision, such as:

- It is hard to understand the grain composition;
- It requires high quality images, so the grains and their details can be easily identified;
- The authors suggest the use of combined learning techniques and a variety of spectral ranges which will detect information that the visual range is unable reach.

Saez-Mas, A. et al. [17] detail a 4-layers architecture and adapt it to two specific case studies. The first case is moving car bodies from the paint plant to the assembly line and the second is analysing a layout of a section used to assemble the engine and transmission set. The 4-layers previously mentioned are:

1. Network Layer—includes the machines, buffers, paths and products to be transformed.
2. Logic Layer—includes decision-making processes that may activate other decision procedures, or even elements in the Network Layer.
3. Database Layer—feeds simulations with the data needed to perform activities and to make decisions. It also stores the results.
4. Visual Layer—eases communication with users and stakeholders.

The architecture promotes separation of the physical system and the logical system, thus allowing a better understanding of the problem, and the reuse of layers in future models that have similarities. It also facilitates exporting the information to be used in statistic tools, because of the Logic and Database layer separation.

### 2.8. Quality Control Systems

The following are quality control systems already available in the market sharing some aspects with our own work.

The European enterprise Qipack (Qipack Official Website: https://www.qipack.com) assures the quality control in packing processes that are based on detailed real-time analysis of each heat sealed product. This system performs a continuous, real-time and non-destructive monitoring, using a high definition camera to record the seal immediately after its production. The system analyzes the pixels received from the camera and search for abnormalities in order to remove products with defect. It is used by industry players such as Capri-Son, Danone, Heineken, Heinz, among others.

TheSealCheck™ (TheSealCheck Official Website: https://www.thesealcheck.com/) is a USA based enterprise is the market leader for thermal-imaging based automated heat seal inspection solutions and presents numerous applications for its software from caps, bags, packages, plastic trays, etc.

Also based in the USA, the PECO-InspX (PECO-InspX Official Website: https://www.peco-inspx.com/) develops solutions for food inspection using mainly X-ray systems. The solution behaviour is similar but it is oriented to detect different defects. The X-ray are not suited to detect lower density objects. The enterprise solutions focus on detecting metal, glass, stone, rubber, and many high-density plastics.

The systems described in the present subsection are commercial, customized systems, and the the accuracy and real-time performance are not made available by the manufacturers to the general public.

## 3. Overview of the Methodology

The methodology proposed allows for the online and continuous assessment of bottle seals, i.e., it is able to inspect and classify the quality of the seals as the bottles are coming out in the conveyor, checking the caps at exit of the thermal camera. The following are the main aspects of the system:

- As bottles are coming out on the production line, thermal pictures of the caps are taken, using an infrared camera mounted above the conveyor belt.
- Single-frame pictures portraying each bottle are extracted. Each frame shows the distribution of heat of the seal inside the cap of a bottle, representing the temperature dispersion of the hot glue used in the sealing.
- After a frame is extracted, it is cleaned to remove noise and binarized. An image signature is then created, through the cumulative sum of the pixels along each row and column. The image signature is then sent to a machine learning-based classifier. Image signatures are explained in more detail in Section 6.2, Figure 7.
- When a defective seal is identified by the classifier, a signal is sent to a mechanical actuator that removes the bottle from the conveyor belt.
- The result of each seal classification is stored in a database, regardless of being defective or not. All pictures analyzed are also stored. This allows for later inspection and retraining the classifier if needed. It also allows for an incremental improvement of the system performance over time.
- Poorly predicted images—either correctly sealed bottles classified as unsealed (False Negatives) or poorly sealed bottles classified as sealed (False Positives)—can be used for retraining of the machine learning model, in order to improve the classifier over time.

The main focus of the present work at this point is the classifier, which is detailed in Section 6. Following are the requirements for a system to support the methodology. An architecture for its implementation is given in the next section.

The main functional requirements of the system are:

- Interact with an infrared camera and process the video output to extract images of individual bottles;
- Process pictures of the bottles to remove noise, binarize and create image signatures with the sum of the rows and columns;
- Store pictures in a database and retrieve them for inspection and retraining of the classifier;
- Control interaction with the classifier to (i) send pictures for classification; (ii) obtain classification results, and (iii) retrain the classifier;
- Interact with the user to control the system, to: (i) start and shut down; (ii) query and manually update the database; (iii) inspect the classifier predictions; and (iv) retrain the classifier;
- Control a mechanical actuator and signal it to remove a bottle with a defective seal.

To enable application of the system to different industrial scenarios, the implementation should be modular and independent from specific hardware vendors. Thus, interaction with the infrared camera and with an actuator should be implemented in modules independent from the database and classifiers.

## 4. Architecture

The architecture of the system was based requirements provided by the specific case-study plant:

- The system should be self-contained in a single computer to minimize space and maintenance requirements;
- All classification decisions must be logged to enable manual inspection for validation and refinement purposes;
- The user interface must me remotely accessible, both inside and outside the plant local network via web browser.

The architecture of the system comprises the following specialized components: the image acquisition module; the classifier module; a database; and lastly the user interface module. All are contained in the same machine, located at the production line. This architecture has the following properties:

- Use a small number of components, in order to reduce the number of equipment and parts needed. A single computer is sufficient to run the software;
- A single machine means that problems are most likely easier to identify and less physical space is needed to store the equipment.
- The communication between modules can all be done inside the computer, which prevents the existence of delays that could make the system miss the removal of an unsealed bottle;
- The architecture is, however, modular and scalable. So the system can be scaled up in order to monitor one or more production lines, or the software modules distributed in order to run in different computers.

**Figure 2:**
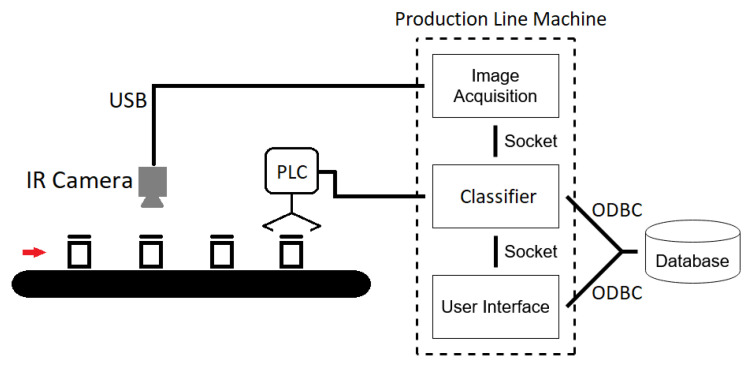
System architecture.

Figure 2 shows a graphical representation of the modular architecture proposed for the system. The four essential software elements are:

1. Image Acquisition module
2. Classifier
3. Database
4. User Interface

The database is logically connected to the Classifier module in order to store the image path and the classification result. It is also connected to the User Interface module, so that the user can verify and manage the prediction results.

In addition to the core software modules, Figure 2 also shows the required hardware components, added to the production line. The hardware comprises an infrared camera, responsible for grabbing the images of the bottles when they are moving through the conveyor-belt, and a programmable logic controller (PLC) responsible for activating the rejection system that removes the unsealed bottles.

A prototype of the system is currently being implemented using the programming language Python. Python was selected because it has extensive libraries supporting Machine Learning and Artificial Intelligence, while, at the same time, providing a simple, short-learning curve development environment.

### 4.1. Image Acquisition

As the bottles are being filled and sealed, they advance one at time through a conveyor-belt, passing through the infrared camera visual range immediately after leaving the thermal camera where the caps are sealed. Thus, when the images are captured the glue that fixes the seals is still hot and the ring of the bottle where the sealant is bound to the bottle opening is clearly visible in the thermal image.

The camera captures images in video mode, and the images are sent via a USB connection to the Image Acquisition module, where they are processed in order to select single frames of each bottle. Only the frames where the seal is clearly visible in the Region Of Interest (ROI) of the image are processed for classification. This also works to detect bottles, without need of using any additional sensors.

These image processing steps are performed mainly using the following Python libraries: Numpy, OpenCV and Skimage. In Section 5.2 the frame selection algorithm is detailed.

The frames selected are stored under a unique name, which follows the template ‘MACHINEID_IMGID_TIME.png’, in order to maintain a log of the relevant images. The ‘IMGID’ is a unique identifier number that will increment for each bottle that is recorded and the ‘TIME’ is the exact time when the frame was saved. With this method it is possible to estimate when a poorly sealed bottle was processed and track it on the production line if needed. The ‘MACHINEID’ is an identifier of the production line where the bottle was processed. That is prepared for future use, in case the system is scaled up to monitor more than one production line at the same time. The frames captured and selected for classification are then sent to the classifier module.

### 4.2. Classifier

The classifier module comprises the classification algorithm and a connection to a database. Once received in this module, an image is classified, in order to decide if the bottle should be removed from the production line (in case it is poorly sealed). This classification is done using a multilayer perceptron implemented in Python using the scipy Sklearn library.

This module is also responsible to emit a signal that will activate a PLC whose function is remove the bottle whose seal was predicted by the classifier as defective. The conveyor-belt moves at a constant velocity, so the time period that a bottle takes to go from the infrared camera visual range to the location where it will be removed is fixed and known, allowing for the activation the PLC to actuate the bottle removal mechanism at the right time to remove the intended bottle. The direct connection between the Server module and the PLC prevents delays in the process and assures the right bottle is removed. The image location and the result of its classification are stored in the database for each image that is sent to the classification module.

### 4.3. User Interface

The web interface has several advantages. One is the reduction of the operator physical presence at the production plant, which is very much in line with the trend experienced in the new Industry 4.0. Through the web interface, any manager or employee can check the logs, make adjustments and train the system no matter where they are in the world, as long as they have access to an Internet connection. That increases people safety and reduces the probability of work accidents, cross contamination or similar problems that may occur in the factory environment. Another advantage is that the web interface facilitates checking the production line at different hours of the day or days of the week.

The user interface allows the user to improve of the classifier at any time. By analyzing the classifications results the users can validate the operations and, in case the neural net made a wrong prediction, they can select that image in order to improve the classifier. This is achieved using the Sklearn MLP Classifier *partial_ fit()* function. This functionality allows this system to continuous learn without having to retrain using the entire dataset.

The use of web-base interface bring the risk of excessive exposure to unauthorized users, in particular from the outside of the plant network environment, creating security concerns. The use of encryption on data transfers, the identification of clients through Secure Sockets Layer/Transport Layer Security (SSL/TLS) protocol used in parallel with a secure database authentication system, and Virtual Private Network (VPN) technology [15] are recommended countermeasures against threats and can be used with our proposed system. Although it is a subject outside the scope of the present paper, we propose the use of those security measures to regulate the access from outside of the company intranet to the system and its user interface, reducing the risk of unauthorized access to data and functionality.

## 5. Image Processing

### 5.1. Data Acquisition

In order to test the algorithms, a total of four videos were grabbed in the production line. While the camera was recording videos, bottles with different sealing defects were put in the conveyor belt and passed through the thermal camera. The camera used was an Optris Pi with an adaptive scale and the resolution of the images obtained is 382 × 289 pixels.

**Figure 3:**
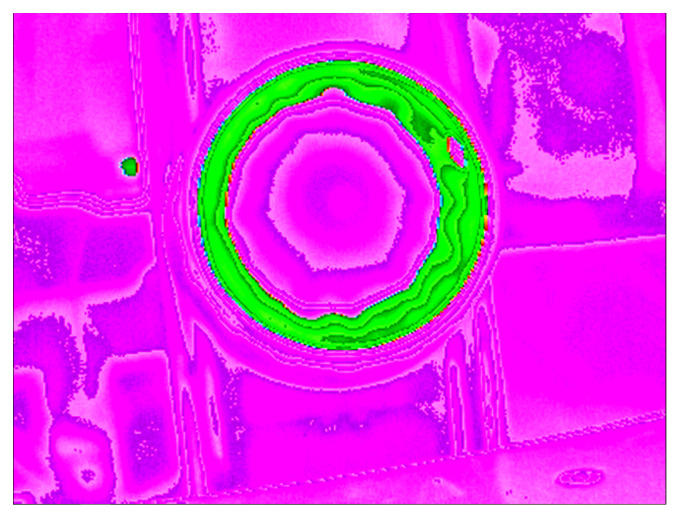
Example of thermal image showing the sealing process done right. In this infrared color scheme, hot locations are represented by the green color while the purple color represents the background environment temperature. The green circle represents the hot area where the seal adhered to the bottle opening.

Figure 3 shows a thermal image of a well sealed bottle. A fully sealed bottle can be identified by a perfect green circle, which appears all around the bottle opening. If the image of the seal obtained has a full circle, of an acceptable thickness, similar to what is shown in the figure, then the sealing is defect-free and the bottle can be moved forward for distribution.

In Figure 3 another hot location can be identified (the small green color dot shown in the picture). This is caused by a motor that is running near to the conveyor belt and in the field of view of the thermal camera. This is the most concerning noise because because it presents itself with the same color as the seal. However, since it is a constant spot, it can be easily identified and removed during image preprocessing. The green circle related to the seal contains a small pink hole in its rim. This hole is a colder area and is caused by a plastic pin that comes in all the bottle caps.However, this has no significant effect on the resulting image signature.

**Figure 4:**
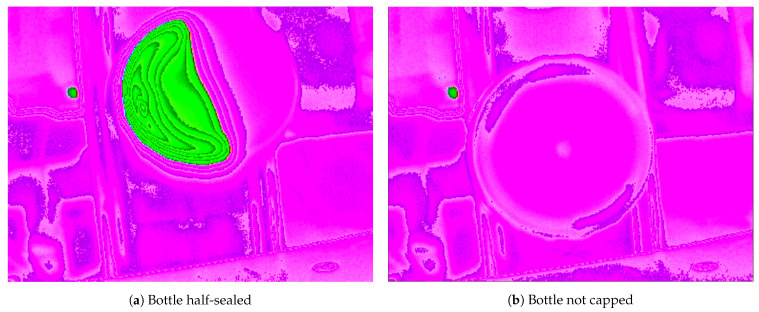
Images of two bottles with problems. The half sealed bottle must be detected and rejected.

Figure 4 shows two examples of results of defective seals. In Figure 4a only half the bottle opening is sealed. The seal inside the cap was warped or cut and did not adhere to the bottle opening as expected. In that situation, the camera still identifies a bottle and in the image obtained it is possible to verify there is only half a circle, representing half the seal. In bottle cap shown in Figure 4b, there is no seal. This situation is different from the first one because the bottle has the same color as the background. It can only be identified because of the faint round shape presented in the image, but this is not sufficient for proper classification using present method. If those bottles appear, they will require additional detection methods such as touch sensors, but this is out of the scope of the present paper.

### 5.2. Image Processing

The frames captured are first manipulated using image processing techniques. As the first step, the best color scheme to work with was chosen. A good color scheme simplifies the noise removal stage.

**Figure 5:**
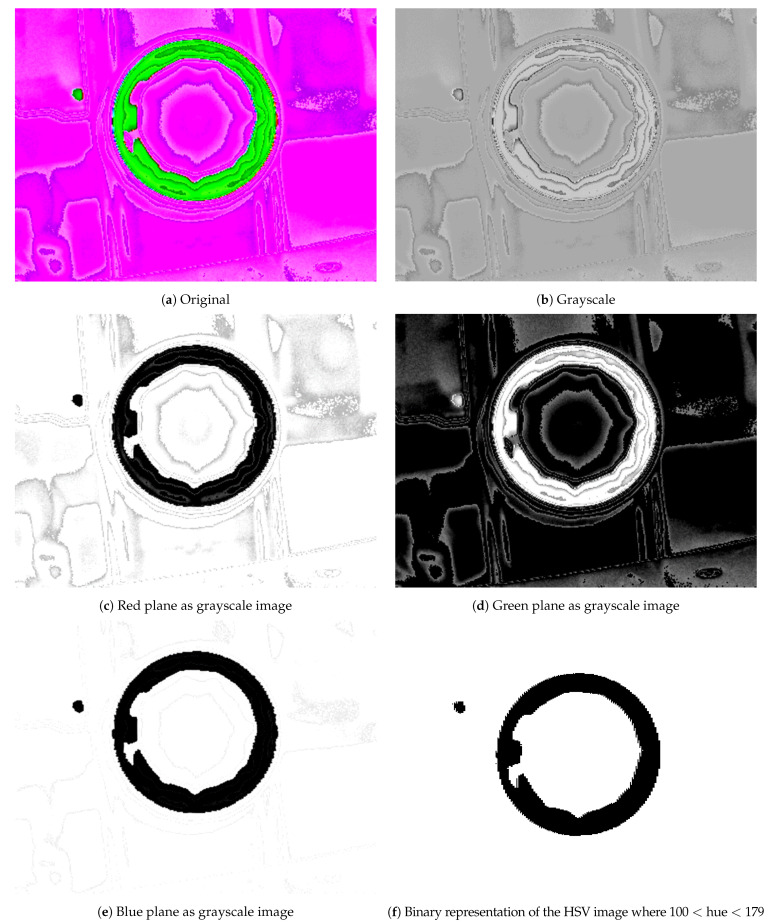
Representation of images using different color schemes.

First, the RGB colour images are converted to the HSV color scheme. HSV is a good representation scheme to highlight the contrast between the seal and the background, as can be seen in Figure 5. It is superior to other image representations in separating signal from noise, as shown in the figure. From all three RGB planes tested (Figure 5c–e), we found good results in the blue plane but there was still noise in the image that needed to be removed using further processing. The HSV color system presented the best results, since the circle is clear and there is very little noise in other areas, as shown in Figure 5f. This was achieved using OpenCV function inRange(), defining an interval of values for hue between 100 and 179 and accepting all saturation and value. It was then possible to isolate the seal presented in the original image. Thus, we selected the HSV color system because it highlighted the seal but also removed most of the image noise.

**Figure 6:**
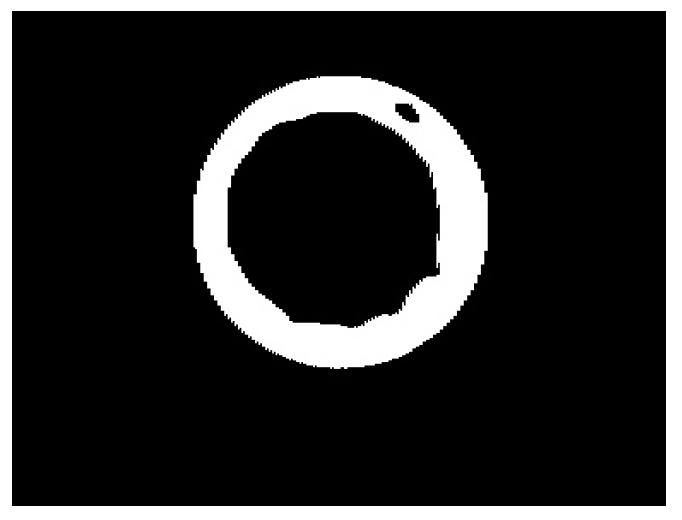
Image of the cap after removing the noise.

After the HSV representation of the image is obtained, the next step is to remove the noise left on the image such as the small black dot presented in Figure 5f. The dot is a reminiscent of the green dot seen in Figure 3, caused by the heat of the motor that is near the conveyor belt and inside the field of view of the thermal camera. The HSV picture is converted to a binary image where white pixels are mapped to value 1 and black pixels are mapped to 0. Then, the area of each group of black pixels is calculated by counting the number of black pixels in the neighborhood, and groups with pixel count lower than a defined threshold are removed from the image, since they are too small to represent any meaningful seal circle. After the steps described above, the image is usually a white frame with a black circle in it representing the bottle seal. Finally, the colors are inverted in order to assure the background value is 0, which simplifies the process of creating the image signature as the sum of the lines and rows. The result will be similar to what is shown in Figure 6.

### 5.3. Frame Selection

The camera frame rate is approximately 25 frames per second. However, the production line serves no more than one bottle every two seconds, and most of the images can be safely ignored.

Only one image of each bottle is necessary for classification. The frame selected must have the seal clearly visible and will then be used to generate the signature that will be input to the neural network.

To determine if a frame is relevant, a Region Of Interest (ROI) was defined, and one line of the ROI is checked for white pixels to determine if it is likely to have a circle or not. A simple but effective method is used to achieve this. A vertical line at mid width of the image is checked pixel by pixel, from the top border of the image and until a quarter of the image height. When a pixel in that line is 1, it means there is a high probability the image has a circle and the frame should proceed for further processing. The next 30 frames are assumed to belong to the same bottle and therefore ignored. If no white pixels are found in that vertical line then the image is very unlikely to contain any relevant information and does not deserve further processing.

### 5.4. Dataset Preparation

For the experiments, four different videos were recorded and used. Table 1 shows the number of bottles that were captured in each video.

The videos contain a total of approximately eight minutes and, in that time, 59 bottles were recorded. Of those, only 56 are detected by the algorithm, as the remainder bottles do not have a cap with seal and are just dummies for testing purposes.

Of the 56 images obtained, 53 are represent correctly sealed bottles and only 3 are related to bottles with sealing problems that should be detected. Therefore, the dataset is small and also unbalanced. To improve the dataset size, we applied a data augmentation technique: all images were rotated using various angles.

Due to the fact that the dataset is not balanced, the number of rotations was adjusted according to the label type:

- Images representing a well sealed bottle were rotated 6 times, increasing 60 degrees per rotation.
- Images representing a poorly sealed bottle were rotated 24 times, increasing 15 degrees per rotation.

Applying smaller angle rotations to images of faulty seals makes sense, since those images are usually not symmetric. Images of well sealed bottles have a higher degree of symmetry, so less is gained when they are rotated.

Once the process was finished, the dataset contained 390 images, 72 of which represented poorly sealed bottles and 318 were well sealed bottles. The unsealed percentage increased from 5.35% to 18.46% of the entire dataset.

## 6. Machine Learning Model

### 6.1. Neural Network

From the numerous machine learning algorithms available, Artificial Neural Networks (ANN) were selected for the present system, considering, among others, the following reasons:

- ANN provide good performance as classifiers when the right parameters are used;
- They are well supported by the Python library Sklearn, which offers simple and effecient methods to implement shallow ANN;
- ANN models are easy to train and adapt, so they can evolve over time if needed, or adapted if the bottle caps change.

The model implemented in our system is the Sklearn Multi-layer Perceptron (MLP) classifier. It learns using LBFGS or stochastic gradient descent (SGD) (MLP Classifier Official Documentation: https://scikit-learn.org/stable/modules/generated/sklearn.neural_network.MLPClassifier.html) algorithms. The LBFGS optimization, however, is not stochastic, so it cannot learn without training the whole dataset. For that reason, the SGD method was used, since it allows the use of the sklearn *partial_ fit()* function that is essential to solve this requirement.

### 6.2. Input Data

After the images are processed and selected as described in Section 5, image signatures are calculated as follows. There are several steps when preparing the images for input to the neural network. The first step is to convert the image to an appropriate size. The original images contain a total of 110,016 pixels, so they are too large to use and can be down-sampled [18]. The higher the quality of the image the easier it is to identify flaws in the sealing process, but down-sampling the image to a size that still keeps most of the information allows for faster processing in real time. The present case uses thermal cameras which already have low resolution, so they were fast to process and were not downsized.

Additionally, instead of using every pixel in each image as an input to the neural network, we computed the sum of white pixels in each row and in each column resulting in two arrays, one for the lines and one for the columns. These arrays are column and row signatures of the binary image. The second array is appended to the first and the resulting vector is the image signature used as input on the neural network.

**Figure 7:**
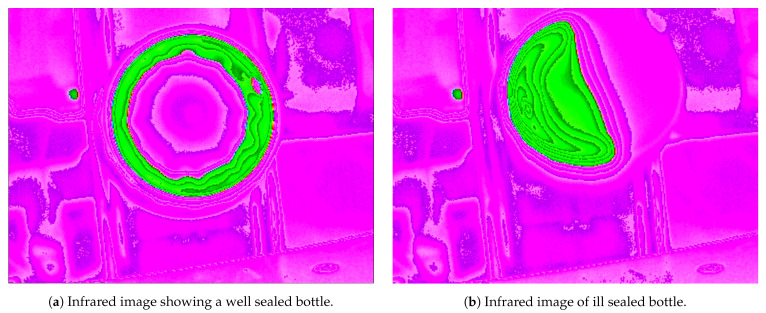

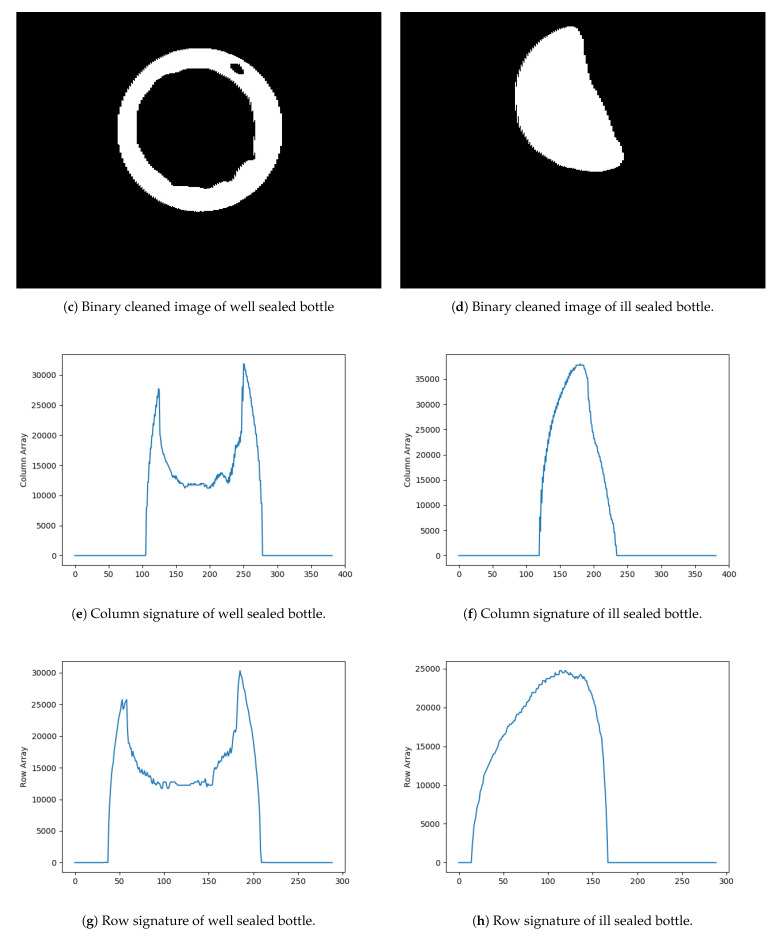
Images obtained during the processing stages of a correctly sealed bottle and a poorly sealed bottle.

Figure 7 shows side-by-side images related to the processing of two bottles. Figure 7a shows a thermal image of a well sealed bottle and Figure 7b shows a thermal image of a poorly sealed bottle. Figure 7c,d are the binary representations of the images, obtained as result of the image processing steps previously explained. Finally, Figure 7e–h are graphical representations of the image signatures. The charts show the vectors obtained by summing the images row-wise and column-wise. The row and column vectors are then appended and used as inputs of the neural network. A correctly sealed image will always present a distribution of pixels similar to the one presented in Figure 7e,g and so, both the row and column signatures will resemble a parable on the middle of the chart. Defective-sealed bottles, however, will have different distributions of the pixels in the binary images and their signatures will differ from the parabolic shapes, as shown in the charts.

### 6.3. Hyper Parameter Optimization

The classifier includes multiple parameters such as hidden layer size, activation function, solver, learning rate and others. Those parameters were optimized using the GridSearch algorithm.

**Figure 8:**
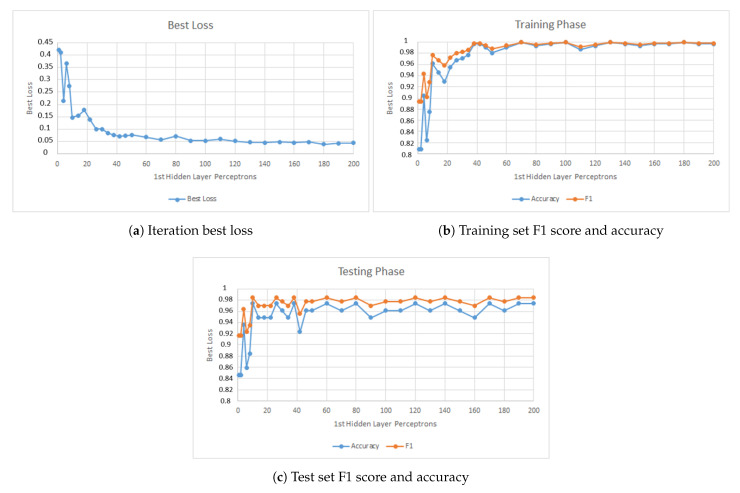
Model behavior when changing the number of perceptrons in the first hidden layer.

The first parameter studied was the number of perceptrons in the first hidden layer. Figure 8 presents results of this phase. Figure 8a shows the model best loss variation when increasing the number of perceptrons in the first hidden layer. Figure 8b shows the model performance when applied to the training set and Figure 8c shows the performance when applied to the test set. The figure shows that above 60 perceptrons the change in the model behavior is small.

When selecting the top values presented in Figure 8c, the *GridSearchCV()* would always select the higher value in range until it capped at 130 perceptrons. However, since the change in the model is small, only 100 perceptrons were used in the final model. The reason that led to this choice is to decrease the chance of overfitting and to reduce the computing power needed to test and train the models in the production line.

**Figure 9:**
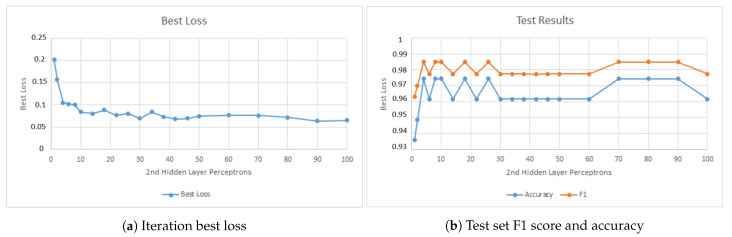
Model performance behavior when changing the number of perceptrons in the second hidden layer.

The second parameter tested was the number of perceptrons in the second hidden layer. Figure 9a presents the model best loss variation and Figure 9b presents the model performance when applied to the test set.

Figure 9b shows that the model accuracy and F1 score are capped around 0.986 and 0.975 respectively. Based on Figure 9a, the value selected was 10 perceptrons since it marks the point where the model loss becomes almost constant.

**Figure 10:**
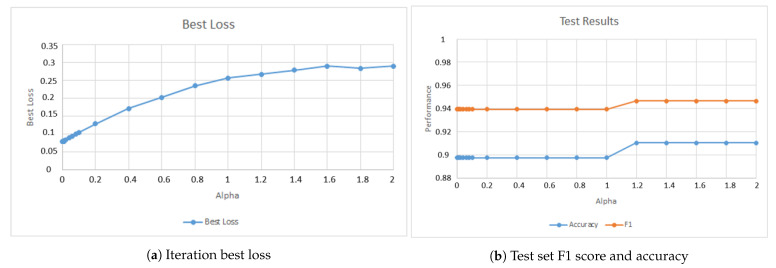
Model performance behavior when changing the value of alpha.

The last parameter tested was the alpha regularization parameter value. Figure 10a shows the model best loss variation and Figure 10b shows the model performance when applied to the test set.

Figure 10b shows the best results when alpha is greater or equal to 1.2. However, the best loss obtained with this value is high, as can be seen in Figure 10a. Once again, the *GridSearchCV()* function was used to select the best value and the result was 1.2.

Up until this point, the MLP Classifier solver, activation function, hidden layer size and alpha are defined. The final tests consist in finding the best learning rate via trial and error.

The inverse scaling learning rate was selected and once again the grid search found the best learning rate.

Despite not being stochastic, LBFGS was also tested and it presented the best results. This happens probably due to the small dataset size that allows the solver to converge faster and perform better as what is mentioned in its official website. If the continuous improvement of the ANN was not a requirement, this solver would be the one selected since it presented good results with only 30 perceptrons in a single hidden layer.

In summary, the neural net parameters are:

- Activation function: logistic sigmoid function;
- Solver: stochastic gradient descent;
- Hidden layer size: (100, 10)
- Alpha: 1.2;
- Learning rate: inverse scaling.

## 7. Tests and Results

The model was trained with 312 images of the augmented dataset. The model was then tested using the test set, which contains the remainder 78 images.

There is one important aspect to consider when creating the neural net and that is, the number of false negatives must be as low as possible. Each time there is a false negative, an unsealed bottle goes to the distribution. In our case study, the bottles contain pesticide which makes it quite dangerous to the public having defective-sealed bottles going to distribution. Therefore, the model was developed with the ability to be learn over time and receive additional train, in a way that false negatives are avoided.

The maximum training iterations was set to 200. However, the results shown in the present paper were obtained with a model that required only 57 iterations in the training phase.

**Figure 11:**
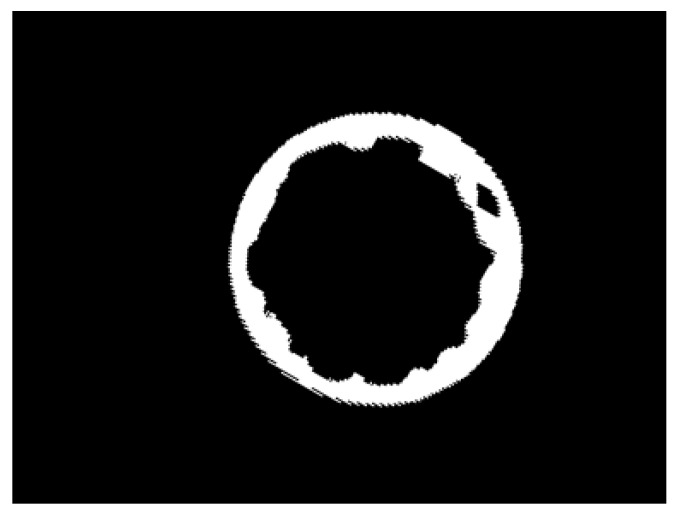
Poorly predicted image by this model in the testing phase.

Table 2 presents the resulting confusion matrix for the model created using the training set. Table 3 presents the results of the same model for the test set. The model performed no errors in the training set but it had a single false negative in the test set that can be seen in Figure 11.

**Figure 12:**
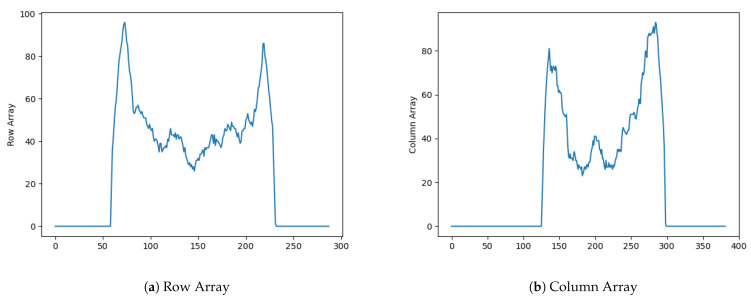
Signature of the incorrectly predicted image.

Figure 11 shows a well sealed bottle that was classified as defectively sealed and would be removed from the production line. The glue layer in this bottle is thinner and more dented than the one in Figure 6 and this may be the cause of this result. The human operator classified it as acceptable, but this is borderline situation considering the thinness and irregularity of the seal. Figure 12a,b show the image row and column signatures. They are very different from the signatures of better sealed bottles as shown in Figure 7f and in Figure 7d.

In the production line implementation, the model would be partial fitted to classify this image correctly and improve its performance. That can be achieved using the partial_fit function provided by the sklearn library, so that the network is trained a number of times until it classifies that particular image correctly.

The model described above has a precision of 98.6% and a recall of 100% for this particular dataset.

Overall, it is a good model, and it can evolve over time, so it satisfies the system requirements. It is a flexible system that can also be trained to deal with different types of bottles or caps, and can be adjusted when the position of the thermal camera needs to be changed.

**Figure 13:**
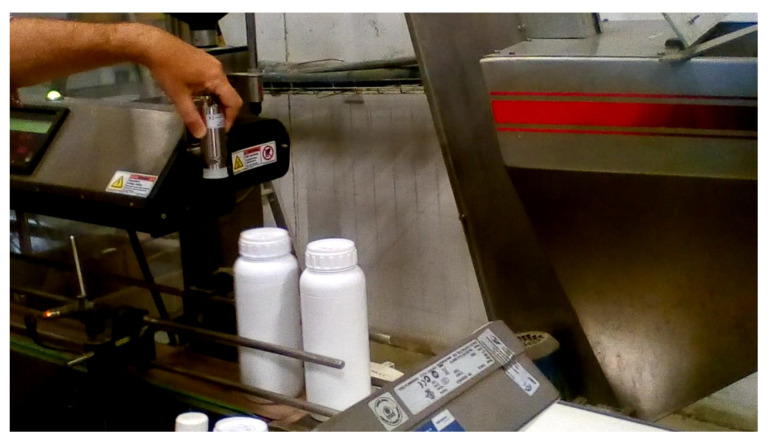
Picture taken in the production line during a stop. It shows two bottles right after they passed below the thermal camera. The human hand is adjusting the thermal camera.

Figure 13 shows a picture of the production line that server as basis for the present work. There are two bottles in the conveyor belt, leaving the thermal chamber. They have just passed below the thermal camera.

## 8. Discussion

The present paper describes the architecture of a system to automate inspection of hot sealed bottles using a neural network which can receive additional train to evolve over time. The system is versatile and can be adjusted to operate with different types of bottles and caps. It is also scalable and can be deployed to serve multiple production lines simultaneously without having to duplicate all the modules.

One limitation of the present approach is that it depends on the image contrast to detect the presence of the cap. Uncapped bottles like the ones presented in Figure 4b will not even be detected since they do not have hot glue on it. They require other sensors and techniques to be detected.

The results show that the system can be trained in a small number of iterations and be evolved to learn specific images.

## 9. Conclusions

Quality control in industrial facilities is very important, to ensure that faulty and possibly dangerous products are detected before they go to consumers. The present paper describes a quality control method using an infrared camera to obtain images, image processing techniques to select and create image signatures, and a machine learning algorithm to classify the images and determine the faulty ones. The machine learning algorithm used is an artificial neural network that uses a stochastic learning method, thus it can evolve and adjust over time. The method does not depend on the detection of specific geometrical shapes, so it can be used to any type of hot seal and bottle. Experimental results show that the method is accurate and fast, thus being appropriate to use in real time.

Future work includes studying the best methods to detect bottles without any seal in them and further develop the user interface.

## Figures and Tables

**Table 1:** General information about the videos recorded.

Video #	Sealed	Unsealed	Skipped	Total
1	15	0	0	15
2	23	1	1	25
3	5	1	0	6
4	11	2	0	13

**Table 2:** Confusion matrix for the training set.

	Actual	Actual
	Positive	Negative
Predicted Positive	60	0
Predicted Negative	0	252

**Table 3:** Confusion matrix for the test set.

	Actual	Actual
	Positive	Negative
Predicted Positive	12	1
Predicted Negative	0	65

## Data Availability

Not applicable.

## References

[1] Asaduzzaman M., Kerschbaumer M., Bodner M., Haman N., Scampicchio M. (2020). Short-wave near infrared spectroscopy for the quality control of milk. J. Near Infrared Spectrosc..

[2] Boiret M., Chauchard F. (2017). Use of near-infrared spectroscopy and multipoint measurements for quality control of pharmaceutical drug products. Anal. Bioanal. Chem..

[3] Pontes R., Mendes M., Farinha J.T., Almeida J. (2019). Motor overheating monitoring using thermal images and artificial neural networks. Proceedings of the 18th International Symposium on Ambient Intelligence and Embedded Systems.

[4] Elgargni M., Al-Habaibeh A., Lotfi A. (2015). Cutting tool tracking and recognition based on infrared and visual imaging systems using principal component analysis (PCA) and discrete wavelet transform (DWT) combined with neural networks. Int. J. Adv. Manuf. Technol..

[5] Haider M., Doegar A., Verma R.K. Fault Identification in Electrical Equipment using Thermal Image Processing. Proceedings of the 2018 International Conference on Computing, Power and Communication Technologies (GUCON).

[6] Zheng G., Wu X., Hu Y., Liu X. Object Detection for Low-resolution Infrared Image in Land Battlefield Based on Deep Learning. Proceedings of the 2019 Chinese Control Conference (CCC).

[7] Dua G., Mulaveesala R. (2018). Thermal wave imaging for non-destructive testing and evaluation of reinforced concrete structures. Insight-Non-Destr. Test. Cond. Monit..

[8] Al-Habaibeh A., Shi F., Brown N., Kerr D., Jackson M., Parkin R.M. (2004). A novel approach for quality control system using sensor fusion of infrared and visual image processing for laser sealing of food containers. Meas. Sci. Technol..

[9] Al-Habaibeh A., Parkin R. (2003). An autonomous low-Cost infrared system for the on-line monitoring of manufacturing processes using novelty detection. Int. J. Adv. Manuf. Technol..

[10] D’huys K., Saeys W., Ketelaere B.D. (2016). Active Infrared Thermography for Seal Contamination Detection in Heat-Sealed Food Packaging. J. Imaging.

[11] Shuangyang Z. Fast Inspection of Food Packing Seals Using Machine Vision. Proceedings of the 2010 International Conference on Digital Manufacturing Automation.

[12] Del Frate F., Pacifici F., Schiavon G., Solimini C. (2007). Use of Neural Networks for Automatic Classification From High-Resolution Images. IEEE Trans. Geosci. Remote Sens..

[13] Parisi L. (2020). m-arcsinh: An Efficient and Reliable Function for SVM and MLP in scikit-learn. arXiv.

[14] Basu S., Das N., Sarkar R., Kundu M., Nasipuri M., Basu D.K. (2012). Handwritten Bangla Alphabet Recognition using an MLP Based Classifier. arXiv.

[15] Dimitris M., Ekaterini V., Milas N., Xanthopoulos N. (2016). A Cloud-based Approach for Maintenance of Machine Tools and Equipment Based on Shop-floor Monitoring. Procedia CIRP.

[16] Vithu P., Moses J. (2016). Machine vision system for food grain quality evaluation: A review. Trends Food Sci. Technol..

[17] Saez-Mas A., Sabater J.P.G., Llorca J.M. (2018). Using 4-layer architecture to simulate product and information flows in manufacturing systems. Int. J. Simul. Model..

[18] Xu Y., Jin Z. Down-Sampling Face Images and Low-Resolution Face Recognition. Proceedings of the 2008 3rd International Conference on Innovative Computing Information and Control.

